# Exploring the utility of RDoC in differentiating effectiveness amongst antidepressants: A systematic review using proposed psychometrics as the unit of analysis for the Negative Valence Systems domain

**DOI:** 10.1371/journal.pone.0243057

**Published:** 2020-12-16

**Authors:** Andrew Hui

**Affiliations:** NorthWestern Mental Health, The Royal Melbourne Hospital, Melbourne, Australia; Temple University, UNITED STATES

## Abstract

**Background:**

RDoC conceptualises psychopathology as neurobiologically-rooted behavioural psychological “constructs” that span dimensionally from normality to pathology, but its clinical utility remains controversial.

**Aim:**

To explore RDoC’s potential clinical utility by examining antidepressant effectiveness through Negative Valence Systems (NVS) domain constructs.

**Method:**

A systematic review was conducted on Web of Science, MEDLINE, EMBASE and PsycINFO for antidepressant trials that included psychometric instruments assessed by Watson, Stanton & Clark (2017) to represent NVS constructs of Acute Threat, Potential Threat and Loss.

**Results:**

221 citations were identified; 13 were included in qualitative synthesis, none for quantitative analysis. All suffered from significant bias risks. 9 antidepressants were investigated, most within 1 construct, and most were found to be effective. Paroxetine, citalopram and fluvoxamine were found to be effective for Acute Threat, fluoxetine, desvenlafaxine and sertraline for Potential Threat, and sertraline, fluvoxamine, fluoxetine and desvenlafaxine effective for Loss. Nefazodone was found to be ineffective for acute fear.

**Conclusion:**

Preliminary evidence supports RDoC NVS constructs’ clinical utility in assessing antidepressant effectiveness, but lack of discriminant validity between Potential Threat and Loss supports their recombination into a single Distress construct. Finding of effectiveness within “normal” construct levels support the utility of a dimensional approach. Testable hypotheses were generated that can further test RDoC’s clinical utility.

## Introduction

The Research Domain Criteria (RDoC) represent the efforts of the National Institutes of Mental Health [1] to address construct validity issues that continue to limit DSM’s ability to drive research into psychopathology [2]. It aims to achieve this through the conceptualisation of psychopathology as distinct “constructs” inspired by behavioural-psychological concepts that could be represented both neurobiologically down different “units of analysis”, dimensionally from normality to pathology, and grouped hierarchically under broad “Domains” [2].

The endeavour is now approaching the end of its first decade, with most research efforts to-date seeking to identify appropriate “elements” for the different fields within the RDoC matrix [3]. Consequently, it is still unclear how RDoC will practically affect future clinical care, although some have envisioned how it could be incorporated in psychiatric assessments [4], whilst others warn of its clinical futility [5].

This systematic review is an attempt to further this discussion by exploring the effectiveness of antidepressants through an RDoC lens. Since the first SSRI was approved by the FDA in 1987, 5 other classes of antidepressants have been released, all sharing the same fundamental pharmacodynamics of enhancing aminergic function [6]. However, the inability to isolate specific biological markers of psychopathology has led to a persistent reliance on psychometric measures that has, with few exceptions, struggled to clearly differentiate amongst individual agent or class in terms of effectiveness amongst various disorders for which they are the first-line psychopharmacologic intervention [7]. Some have argued that contributing to this problem is an overreliance of psychopharmacologic research on psychometrics that rely excessively on symptom lists derived from diagnostic criteria [8]. Therefore, psychometrics that are sensitive to the phenomenological manifestations of a new paradigm of pathophysiology with clearer biological underpinnings should allow for clearer descriptions of the clinical effectiveness of antidepressants.

The DSM disorders for which antidepressants are the first-line psychopharmacological agents correspond best to disturbances within the RDoC Negative Valence Systems (NVS) domain constructs of “Acute Threat / ‘Fear’ “, “Potential Threat / ‘Anxiety’”, “Sustained Threat”, “Frustrative Nonreward”, and “Loss” [9]. Whilst the RDoC Negative Valence Domain workgroup had identified the conceptual underpinnings of these constructs, there was a consensus that “additional efforts should be targeted to develop better [self-report] measures”. In response, Watson, Stanton and Clark [10] critiqued a number of psychometric instruments based on their convergent and discriminant validity with respect to these constructs, including the ones listed by the Workgroup. Consequently, they proposed the following measures:

- Acute Threat:
  ○ Fear Questionnaire [11]: Agoraphobia or Blood Injury phobia subscales
- Potential Threat:
  ○ Profile of Mood States (POMS) [12]: Tension subscale,
  ○ Positive and Negative Affect Scale–Extended Form (PANAS-X) [13]: Fear subscale,
  ○ NEO Personality Inventory 3 (NEO-PI-3) [14]; Anxiety subscale,
  ○ Temperament and Affectivity Inventory (TAI) [15]: Anxiety subscale, and
  ○ Faceted Inventory of the Five Factor Model (FI-FFM) [16]: Anxiety subscale
- Loss:
  ○ POMS: Depression subscale,
  ○ PANAS-X: Sadness subscale,
  ○ TAI: Depression subscale,
  ○ FI-FFM: Depression subscale, and
  ○ NEO-PI-3: Depression subscale

They were not able to propose any instruments that adequately reflect the Sustained Threat and Frustrative Nonreward constructs. Whilst some antidepressants have also been used for indications that correspond with disturbances within other RDoC constructs, such as Positive Valence Systems (e.g. bupropion and nicotine dependence), Cognitive Systems (e.g. atomoxetine and ADHD), Arousal and Regulatory Systems (e.g. trazodone and insomnia), and Sensorimotor Systems (e.g. duloxetine and chronic pain), these are exceptions rather than the norm. For the purposes of drawing distinctions amongst antidepressants based on their effectiveness to treat disorders they are primarily indicated for, and to explore the utility of the RDoC conceptualisation of such disorders, this review will thus focus on the effectiveness of current generation antidepressants on the NVS using psychometric measures proposed by Watson, Stanton & Clark [10].

## Methods

A search of the literature using the MEDLINE, Web of Science, Embase and PsycINFO databases was performed in September 2019 to identify relevant studies. Inclusion criteria were: clinical trials with at least one subject group where the intervention consists of only one antidepressant of an SSRI or later generation (i.e. serotonin and noradrenaline reuptake inhibitors, serotonin modulators and stimulators, serotonin antagonists and reuptake inhibitors, noradrenaline reuptake inhibitors, noradrenaline dopamine reuptake inhibitor), antidepressant must be administered at a dose and frequency considered to be therapeutic (daily for at least 4 weeks), and outcomes include psychometric measures using at least one of the subscales listed above.

Exclusion criteria were concurrent biological agent administration. The decision was made not to exclude certain populations (e.g. children, elderly, organic illness) in order to assess the transdiagnostic, dimensional applicability of the RDoC paradigm. The decision was also made not to mandate placebo-controls, blinding or a follow-up period of at least 6 months in order to allow more relevant papers to be included for analysis, since the context of this review is to further discussions about the potential utility of RDoC.

The following search strategy was used in the ‘topic’ field of Web of Science (operated by Clarivate Analytics), and the ‘abstract’ field of MEDLINE, EMBASE and PsycINFO (operated by Ovid): (‘citalopram’ OR ‘escitalopram’ OR ‘paroxetine’ OR ‘fluoxetine’ OR ‘fluvoxamine’ OR ‘sertraline’ OR ‘desvenlafaxine’ OR ‘venlafaxine’ OR ‘levomilnacipran’ OR ‘milnacipran’ OR ‘duloxetine’ OR ‘vilazodone’ OR ‘vortioxetine’ OR ‘nefazodone’ OR ‘trazodone’ OR ‘reboxetine’ OR ‘teniloxozane’ OR ‘viloxozane’ OR ‘bupropion’) AND (‘PANAS’ OR ‘Positive and Negative Affect Scale’ OR ‘Profile of Mood States’ OR ‘POMS’ OR ‘TAI’ OR ‘Temperament and Affectivity Inventory’ OR ‘NEO Personality Inventory’ OR ‘NEO-PI-3’ OR ‘FI-FFM’ OR ‘Faceted Inventory of the Five Factor Model’ OR ‘FQ’ OR ‘Fear Questionnaire’).

The decision was made to use names of individual antidepressants, and to use the “abstract” field rather than “keyword” field in MEDLINE, EMBASE and PsycINFO in order to increase the sensitivity to articles that were not originally designed to answer the question posed by this systematic review. The “topic” field in the Web of Science database was chosen for the same reason because it searches the abstract in addition to keywords.

The results from MEDLINE, EMBASE and PsycINFO were de-duplicated using Ovid’s built-in function. Both platforms’ citations were exported into the EndNote X8 referencing software, and any duplicates arising from overlap between the two platforms removed using the software’s built-in function. The abstracts were examined to remove citations that did not meet inclusion criteria. Where it was not clear from the abstract, the full-text was obtained and the methods examined to determine whether it met the inclusion criteria. The reason each article was excluded was documented.

For all included studies, the following information was extracted into a table: antidepressant investigated (including dose range, duration and mechanisms of ensuring treatment integrity), authors, DSM diagnoses (including comorbidities in sample, and method of ascertaining diagnoses), medical comorbidities and concurrent treatments (including method of ascertaining diagnoses), outcome measure of interest used (including times of measurement, and other outcome measures used in study), study design (including method of recruitment, randomisation, blinding, control groups), participants in each intervention arm (including numbers started and completed trial, how those who didn’t complete trials were treated, and results for outcome measures of interest (including confidence intervals, statistical significance, p values). Where the study compared an antidepressant against placebo, standardised mean differences (SMD) was derived from the change scores provided adequate data about associated estimates of variance was reported (e.g. standard deviations, confidence interval, standard errors).

Risk of biases was analysed for each study depending on the presence of randomisation; randomised studies were analysed using the methodology and criteria as specified by the Cochrane Risk of Bias Tool for Randomized Trials (RoB2) [17], whilst non- or quasi-randomised studies will be analysed using the methodology and criteria specified by the Risk of Bias in Non-Randomized Studies—of Interventions (ROBINS-I) tool [18].

In instances where more than one study investigated the same antidepressant with identical outcome measures of interest, a meta-analysis of their results was considered. The key factor would be a priori homogeneity amongst the samples in each study. Given the transdiagnostic interest of this systematic review, this did not mean similar primary diagnoses, but rather similar control groups, dose ranges, duration of antidepressant treatment, or baseline scores. Other important contingent factors included reporting of standard deviations or errors and p values.

If more than 10 studies were included in a meta-analysis, publication bias would be assessed based on funnel plot asymmetry. The extent of statistical heterogeneity would be assessed by calculating I2 for each medication-psychometric outcome. The nature of statistical heterogeneity would be investigated through appropriate subgroup analysis or meta-regression; the former would be used for categorical study characteristics and the latter for continuous study characteristics. Likely study characteristics that could be effect modifiers include dosage, duration of medication, presence of blinding and use of randomisation, nature of control intervention, and use of randomisation. All statistical analyses and graphing would be performed using RevMan Manager 5.3 software published by the Cochrane Collaboration

This review protocol had not been previously published.

## Results

**Fig 1:**
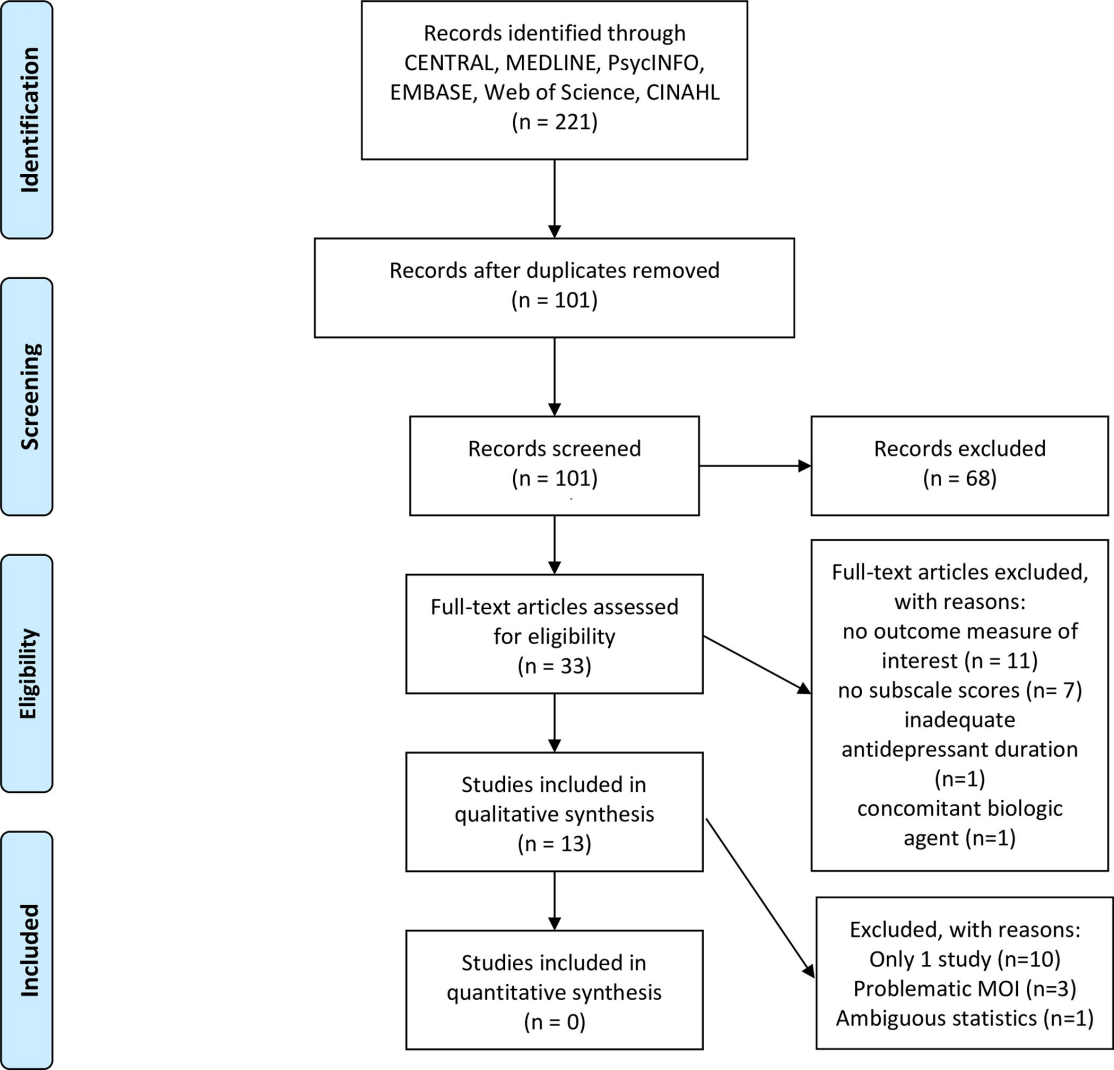
PRISMA flow diagram of the systematic review. (MOI: measure of interest).

185 citations were identified, of which 173 were found not to meet the criteria for inclusion; consequently, 13 articles were included for the systematic review (Fig 1). The majority of citations excluded were due to their lack of an antidepressant interventional nature (e.g. review article, antipsychotics) or their lack of an outcome measure of interest. In fact, these constitute the vast majority of citations whose abstracts did not contain the required search terms; all these abstracts were from the Web of Science database, due to the “topic” field searching both “keyword” as well as “abstract”. Table 1 outlines the relevant properties of the included studies, and Tables 2 and 3 outline the areas of potential biases.

**Table 1:** Properties of studies included for analysis.

Antidepressant (including dose range, duration, adherence)	Authors	Psychiatric diagnoses and comorbidities (including method of identification)	Medical comorbidities and concurrent treatments (including method of identification)	Outcome Measures (including times of measurement)	Number of Subjects (including at commencement, completion, and analysis)	Results of Outcome Measures (including relevant confidence intervals, standard deviations, and/or p values)
• Citalopram (20-50mg, 2 months) • Paroxetine (20-50mg, 2 months)	Perna, Bertani [19]	• Panic disorder +/- agoraphobia (DSM-IV; clinical interview + MINI) • Nil psychiatric comorbidities (physical examination and medical history assessment)	• Nil significant physical illnesses (history and examination) • Nil concurrent psychotropic meds or psychotherapy	• FQ (Baseline, days 7 & 60) Others: • Panic Associated Symptom Scale • Sheehan Disability Scale (SDS)	• Citalopram: 30 started, 27 completed; 27 analysed • Paroxetine: 28 started, 25 completed; 25 analysed	FQ Agoraphobia • Significant reductions from baseline to endpoint according to post-hoc comparisons (p not stated): • Citalopram: baseline 15.7 +/- 12.3; endpoint 9.9 +/- 9.1 • paroxetine baseline 15.6 +/- 11.9; endpoint 8.4 +/- 10.4 • Significant time effect found for antidepressant treatment (F 3.8, p<0.05) FQ Blood injury • Significant reductions from baseline to endpoint according to post-hoc comparisons (p not stated): • Citalopram: baseline 21.1 +/- 10.8; endpoint 15.6 +/- 9.9 • Paroxetine baseline 17.5 +/- 9.5; endpoint 12.4 +/- 10.1 • Significant time effect found for antidepressant treatment (F 9.0, p<0.001)
• Desvenlafaxine (100mg or 150mg, 12 weeks)	Cheng, DuPont [20]	• Nil GAD, mood & psychotic disorders (DSM version not stated; method not stated)	• Vasomotor symptoms post-menopause • Nil concurrent physical illnesses • Nil concurrent psychotropic or hormonal meds	• POMS (Baseline, weeks 4 & 12) Others: • Greene Climacteric Scale • Menopause Symptoms–Treatment Satisfaction Questionnaire	• 100mg: 153 started, 134 completed; 120 analysed • 150mg: 152 started, 137 completed; 117 analysed • Placebo: 153 started, 138 completed; 123 analysed	Significant reductions in POMS Tension and POMS Depression vs placebo (p<0.05):POMS Tension • 100mg: Baseline 9.3 (SD = 6.7), change -4.1 +/- 0.4 • 150mg: Baseline 9.0 (SD = 7.2), change -3.9 +/- 0.4 • Placebo: Baseline 8.6 (SD = 6.4), change -2.4 +/- 0.4 • No statistically significant difference between doses (p value not stated) POMS Depression • 100mg: Baseline 8.3 (SD = 8.6), change -5.5 +/- 0.6 • 150mg: Baseline 8.2 (SD = 11.1), change -4.5 +/- 0.6 • Placebo: Baseline 7.4 (SD = 9.6), change -2.3 +/- 0.6 • No statistically significant difference between doses (p value not stated)
• Escitalopram 10-20mg, 12 weeks	Alamy, Wei [21]	• Specific phobia (DSM-IV as per MINI) • Nil comorbid MDD, GAD, Social Phobia, OCD, PD, PTSD (method not stated) • Nil recent history of substance use/dependence (method not stated) • Nil lifetime BD, SCZ, organic brain syndrome (method not stated)	• Nil significant abnormalities as per haematology, chemistry, serum pregnancy (for women) & ECG • General direction for subjects to make efforts to expose themselves to avoided situation	• FQ (Screening, baseline, weeks 1,2,4,8 and 12) Others: • Main Phobia Scale (MPS) • CGI-I • HAM-A • MINI phobia module • Symptom Occurrence Scale	• Escitalopram: 6 started, 5 completed; 5 analysed • Placebo: 7 started, 6 completed; 6 analysed	No statistically significant reduction in FQ found (p not stated):FQ Agoraphobia: • Escitalopram: Baseline 3.0 (SD = 2.4), endpoint: 1.2 (SD = 1.1) • Placebo: baseline 2.9 (SD = 4.3), endpoint: 0.4 (SD = 0.8) FQ Blood Injury: • Escitalopram: Baseline 4.8 (SD = 4.1), endpoint: 1.6 (SD = 1.3) • Placebo: Baseline 6.9 (SD = 8.3), endpoint: 4.0 (SD = 7.2)
• Fluoxetine (20mg, 6 months)	Wood, Mortola [22]	• Late Luteal Phase Dysphoric Disorder (SCID for DSM-III-R) • Comorbidities excluded (SCID for DSM-III-R and psychometric abnormalities during follicular phase: - BDI > = 12 - STAI > = 45 - MMPI T-score > = 70 - POMS total T-score > = 70)	• Premenstrual Syndrome (history, examination, Calendar of Premenstrual Experiences) • Nil significant medical or gynecologic disorders (history and examination) • Nil comments on concurrent meds	• POMS (Baseline, months 3 & 6) Others: • Calendar of Premenstrual Experiences • Beck Depression Inventory (BDI) • State-Trait Anxiety Inventory (STAI) • Minnesota Multiphasic Personality Inventory	• 8 started, 8 completed, 8 analysed	Significant differences between luteal phase and follicular phase when baseline T-scores of fluoxetine and placebo groups pooled (p<0.01) (individual group baselines not stated, nor was the sample from which the T-scores were derived described): • POMS Depression T-score: Luteal: 59.6 +/- 4.6 Follicular: 44.8 +/- 5.2 • POMS Tension T-score: Luteal: 56.6 +/- 3.3 Follicular: 40.5 +/- 3.6 Luteal phase: significant differences between fluoxetine and placebo (p<0.005) • POMS Depression T-score: Fluoxetine: 39.7 +/- 2.9 Placebo: 50.9 +/- 4.5 • POMS Tension T-score: Fluoxetine: 39.1 +/- 3.4 Placebo: 50.4 +/- 3.8 Follicular phase: no significant differences between placebo & fluoxetine (p not stated): • POMS Depression T-score: Fluoxetine: 39.7 +/- 2.2 Placebo: 43.4 +/- 2.9 • POMS Tension T-score: Fluoxetine: 37.8 +/- 2.9 Placebo: 43.1 +/- 3.7
• Fluvoxamine (150mg, 12 weeks)	Sharp, Power [23]	• Panic disorder +/- agoraphobia (DSM-III-R; clinical interview + HAM-A > = 15) • Nil depressive disorder (MADRS > = 21) • Nil OCD, paranoid PD, psychotic, manic or substance disorders (method not stated)	• Nil severe illnesses (as per GP referral) • Nil concurrent or recent psychotropics • Nil past psychotherapy	• FQ (baseline, weeks 7 & 12, 6 months) Others: • Hamilton Anxiety Scale • Montgomery-Asberg Depression Rating Scale (MADRS) • Panic diaries (created by investigators)	• Fluvoxamine: 36 started, 24 completed; 29 analysed • Placebo: 37 started, 20 completed; 28 analysed	FQ Agoraphobia • Fluvoxamine: Significant difference found vs placebo at day 84 (p<0.05) • Fluvoxamine: Significant reductions between baseline and endpoint found, t = 3.44, df = 28, p<0.002 (but baseline and end-point scores not stated) • Fluvoxamine: Significant time and interaction effects found
• Fluvoxamine (50-200mg*, 4 weeks; *study stated a maximum range of “209mg” but this was likely a typographic error)	Itil, Shrivastava [24]	• Major affective disorders (RDC; clinical interview + HAMD > = 15) • Nil substance dependence (method not stated)	• Nil significant organic disease • Nil concurrent or recent psychotropics	• POMS (baseline, weeks 1,2,3 and 4) Others: • Clinical Global Impression [25] • Hamilton Depression Scale (HAM-D) • SCL-90 • BDI • Dosage record • Treatment Emergent Signs & Symptoms	• Fluvoxamine: 22 started, 10 completed; 9 analysed • Imipramine: 25 started, 13 completed; 14 analysed • Placebo: 22 started, 11 completed; 14 analysed	POMS Depression • Significant difference between fluvoxamine vs placebo at week 3 (p = 0.028) but not at week 4 (p = 0.06)
• Nefazodone (300-600mg, 12 weeks; although results indicated final dosage range was 200-600mg; disparity not discussed)	Van Ameringen, Mancini [25]	• Social phobia–generalised (DSM-IV; SCID-I/P) • Nil exclusions (sample included MDD, dysthymia, PD with agoraphobia, OCD, alcohol abuse/dependence)	• Nil recent or concurrent psychotropics • Nil current psychotherapy • Concurrent organic illnesses not discussed	• FQ (baseline, weeks 4, 8 & 12) Others • BDI • STAI • SDS • Social Adjustment Scale Self-Report (SAS-SR) • Fear of Negative Evaluation Scale (FNES) • Social Avoidance and Distress Scale (SADS) • Social Anxiety Thoughts Questionnaire (SATQ) • Brief Social Phobia Scale (BSPS) • Liebowitz Panic and Social Phobic Disorders rating form (LPSPD)	• 23 started, 21 completed, all evaluated	FQ-Agoraphobia • No significant difference between baseline (9.0, SD = 7.8) and week 12 (6.4, SD = 6.5), F2.3, df 3,51, p = 0.09.
• Paroxetine (10-60mg, 12 weeks)	Mancini and Ameringen [26]	• Social phobia–generalised (DSM-III-R; SCID-I/P) • Nil exclusions (sample included MDD, dysthymia, PD, agoraphobia, PD with agoraphobia, simple phobia, OCD, GAD, alcohol abuse/dependence)	• Nil recent or concurrent psychotropics • Nil current psychotherapy • Concurrent organic illnesses not discussed	• FQ (baseline, weeks 4, 8 & 12) Others • BDI • STAI • SDS • SAS-SR • FNES • SADS • SATQ • LPSPD	• 18 started, all completed	FQ-Agoraphobia • Significant difference between baseline (11.8 +/- 10.8) and week 12 (5.8 +/- 7.1), F3.8, df 3,39, p = 0.017
• Sertraline (50-150mg, 12 weeks)	Alpert, Silva [27]	• MDD (DSM-III-R; semi-structure interview including DSM-III-R checklist; severity HAMD > = 18) • MMSE > = 23 • Nil other Axis I diagnoses (semi-structured interview)	• Nil acute or unstable physical conditions • Concomitant temazepam or chloral hydrate PRN allowed • Nil other recent or concurrent psychotropics	• POMS (Baseline, week 12) Others • Folate • HAM-D	• Sertraline: 12 started, 12 completed, 12 analysed • Nortriptylline: 10 started, 10 completed, 10 analysed	POMS Depression • Sertraline: Change from baseline (1.5, SD = 0.67) to endpoint (1.2, SD = 1.13) not statistically significant (p value not stated)
• Sertraline (50mg, 100mg, 200mg; 6 weeks)	Fabre, Abuzzahab [28]	• MDD (DSM-III; unclear diagnostic method; severity HAMD > = 22) • Nil history of psychotic disorders or current substance abuse (method not stated)	• Nil history of significant medical disease • PRN chloral hydrate allowed except for “nights before psychiatric scale testing” • Nil recent or other concurrent psychotropics	• POMS (Baseline, weeks 1 to 6) Others • HAM-D • CGI • ECG • Vitals • FBE • Biochemistry • Urinalysis	• 50mg: 95 started, 59 completed; 90 “all-patients group” (those who took medication on or after 11^th^ day), 82 “evaluable group” (those who took 1+ dose of medication) • 100mg: 92 started, 47 completed; 89 “all-patients group”, 75 “evaluable group” • 200mg: 91 started, 39 completed; 82 “all-patients group”, 56 “evaluable group” • Placebo: 91 started, 86 “all-patients group”, 46 completed; 76 “evaluable group”	Results for “all-patients group”:POMS Depression • Sertraline 50mg: baseline 2.8 +/- 1.1; change vs endpoint -0.5 +/- 1.1 significantly different vs placebo, p = < 0.01 • Sertraline 100mg: baseline 2.7 +/- 1.0; change vs endpoint -0.5 +/- 1.1 statistically different vs placebo, p = <0.05 • Sertraline 200mg: baseline 2.5 +/- 0.7; change vs endpoint -0.4 +/- 1.0 statistically different vs placebo, p = <0.05 • Combined: baseline 2.7 +/- 1.1; change vs endpoint -0.5 +/- 1.0 statistically different vs placebo, p = <0.01 • Placebo: baseline 2.6 +/- 1.1; change vs endpoint -0.1 +/- 0.7 • No statistically significant differences amongst different dosages (p value not stated).
• Sertraline (50-200mg, 8 weeks)	Lydiard, Stahl [29]	• MDD (DSM III-R; Clinical interview; severity HAMD > = 18) • Nil concurrent dysthymia, bipolar, severe GAD, OCD, PTSD, psychotic disorders, severe PDs, substance dependence (Clinical interview)	• Nil concurrent significant medical illness (history, examination, ECG & blood tests) • Temazepam or chloral hydrate PRN allowed • Nil concurrent psychotropics	• POMS (baseline, weeks 1 to 8) Others • BDI • MADRS • Global Assessment Scale • CGI • Quality of Life Enjoyment and Satisfaction Questionnaire • Health-Related Quality of Life battery, v2	• Sertraline: 132 started, 96 completed, 128 analysed • Amitriptyline: 131 started, 81 completed, 127 analysed • Placebo: 129 started, 92 completed, 125 analysed	POMS Depression • Sertraline: change from baseline 1.9 (SE = 0.08) to endpoint by -1.0 (SE = 0.08) statistically different, p<0.005 • Placebo: change from baseline 1.9 (SE = 0.08) to endpoint by -0.5 (SE = 0.08) statistically different, p<0.005 • Change in POMS-D between sertraline and placebo was significantly different, p<0.001
• Sertraline (50,150mg, 12 weeks)	Finkel, Richter [30]	• MDD (semi-structured interview that included DSM-III-R checklist; severity HAMD24 > = 18) • Excluded: Nil acute or chronic organic mental disorder, MMSE <24, “clinically significant psychiatric illness, active suicidality”	• Hypnotics allowed • Nil acute or unstable medical condition or Ix abnormalities (physical exam, history, ECG, lab investigations) • Nil concurrent anticoagulants (except aspirin) • Nil current or past history of neurological disease • Nil past non-response to 6+ weeks of 2+ adequate doses of antidepressants	• POMS Others • HAM-D • HAM-A • CGI-S/I • Q-LES-Q • MMSE • DSST • SLT • Plasma drug concentrations	• Sertraline: 39 started, 26 finished; 7 quit due to SE; none due to lack of efficacy mean dose 100 +/- 40mg; serum levels not stated • Nortriptylline: 37 started, 19 finished; 11 quit due to SE; none due to lack of efficacy; mean dose 70+/-30mg; 29% had serum levels <50ng	No significant difference in concomitant meds or baseline valuesPOMS Tension • baselines: not stated • change from baseline: -5.9, significantly different vs nortriptyline • time effect not factored POMS depression results not stated
• Venlafaxine (week 1: 75mg, weeks 2–8: 150mg, 8 weeks)	Ozdemir, Boysan [31]	• 1^st^ episode MDD (DSM IV-TR; SCID-I) • Nil current or past history of bipolar disorder or substance dependence (method not stated)	• Concurrent illnesses or medications not discussed	• POMS (baseline, weeks 1,2,4 & 8) Others • HAMD • BDI	• Venlafaxine: 25 started, all completed • Venlafaxine + Bright Light Therapy: 25 started, all completed	POMS Depression • Venlafaxine: baseline 37.16 +/- 11.36; endpoint 11.12 +/- 9.11; statistically significant reduction over time, p<0.001 POMS Tension • Venlafaxine: baseline 23.68 +/- 4.88; endpoint 9.60 +/- 3.93; statistically significant reduction over time, p<0.001

**Table 2:** Risk of bias table of included randomised studies, using revised Cochrane risk-of-bias tool for randomized trials (RoB 2) [17].

Study	Domains of Analysis					
	Randomisation	Unintended deviation from intervention	Missing outcome data	Outcome measurement	Selective reporting	Overall Rating
Perna, Bertani [19]	**High risk**	**High risk**	**Low risk**	**Some concerns**	**Low risk**	**High risk**
	Allocation sequence known to investigator	Neither subjects or participants blinded, and adherence not assessed or possible non-adherence factored in to statistical analysis	Vast majority of subjects included in mITT analysis with only 1 participant in each group (n = 30 and 28) excluded due to potential side effects	Subjects not blinded, although unlikely self-report would be influenced by knowledge of intervention as both groups are active antidepressants and similar low rates of side effects reported	Results analysed according to pre-specified plan	
Cheng, DuPont [20]	**Low risk**	**High risk**	**High risk**	**High risk**	**High risk**	**High risk**
	Allocation sequence concealed by software, and baseline characteristics between groups not significantly different	Both participants and investigators blinded; however, despite adherence being assessed, no discussion of impact of non-adherence on study participation or statistical analysis, and actual adherence not reported; moreover, final numbers analysed less than numbers completing study, but reasons for this not stated or discussed	Final numbers analysed less than numbers completing study–this was neither stated or discussed, so likelihood that data missed could have affected true value cannot be ruled out	Subjects poorly blinded, as participants could infer desvenlafaxine status based on side effects, which was significantly more than placebo during rapid uptitration; moreover, subjects in 150mg group2 could infer this as no indication 150mg was specially formulated to be available in 1 tablet, as was 100mg and placebo	Results not analysed according to pre-specified plan, although all scores were presented	
Alamy, Wei [21]	**Some concerns**	**High risk**	**Some concerns**	**High risk**	**Some concerns**	**High risk**
	Concealment of allocation not discussed, nor baseline characteristics of individual groups reported although lack of statistical differences stated	Neither subjects or investigators blinded; also, adherence not assessed or possible non-adherence factored in to statistical analysis	1 out of 7 placebo participants dropped out at week 2 but was not included in analysis, despite pre-planned protocol that includes all who returned for at least 1 post-baseline review commencing at week 1 for mITT analysis; however, since reason given was not efficacy but “personal”, unlikely to have influenced true value	Subjects not blinded	Only endpoint scores presented even though change in scores analysed as per pre-specified plan; however, reported results were not statistically significant	
Wood, Mortola [22]	**Some concerns**	**High risk**	**Low risk**	**Low risk**	**High risk**	**High risk**
	Concealment of allocation not discussed, nor baseline numbers or characteristics of each group stated, although analysis did factor in order of cross-over	Both subjects and investigators blinded; however, adherence not assessed or possible non-adherence factored in to statistical analysis	No missing outcome data	Subjects blinded, and lack of statistically significant differences in adverse effects between groups mitigates disproportionate risk of inference of treatment status	Baseline characteristics of individual starting groups during first period not stated, and no baseline characteristics prior to commencement of second period also not stated; POMS raw scores transformed into T-scores without reporting raw scores, or describing characteristics of sample from which T scores were derived	
Sharp, Power [23]	**Some concerns**	**Low risk**	**High risk**	**High risk**	**High risk**	**High risk**
	Concealment of allocation sequence not discussed; no baseline characteristics reported although lack of statistical differences stated	Both subjects and investigators blinded, with adherence monitored by return pill counts and those with “concerns about adherence” excluded	Data that were excluded from analysis likely included true efficacy of antidepressant, e.g. those who reported lack of efficacy, started concurrent psychotropics, or developed alcohol abuse	Subjects blinded to medication, but given study included treatment arms that combined medication with psychotherapy, and those on medication only were aware that they were only given therapeutic engagement, it is likely that their self-report was influenced by this knowledge	No pre-specified plan of statistical analysis discussed, nor baseline or change scores reported	
Alpert, Silva [27]	**High risk**	**Low risk**	**Low risk**	**Low risk**	**Some concerns**	**High risk**
	Concealment of allocation sequence not discussed, nor baseline demographics reported although imbalance in gender acknowledged; different numbers of participants in each group, with method of randomisation not stated	Both subjects and investigators blinded; adherence monitored by pill-count with <75% leading to termination from study	No missing outcome data	Subjects blinded well with number of pills administered and manner of uptitration factored in; side effects not discussed but unlikely to lead to inference of treatment group as both groups were antidepressants	Analysis much more in-depth than in pre-specified plan	
Fabre, Abuzzahab [28]	**High risk**	**High risk**	**Low risk**	**Low risk**	**Low risk**	**High risk**
	Concealment of allocation sequence not discussed, with appearance of significant differences amongst groups at baseline; significance testing not done	Both subjects and investigators blinded; however, adherence not assessed or possible non-adherence factored in to statistical analysis	Whilst almost 50% of participants discontinued, proportion similar amongst the groups, and most subjects included in mITT analysis	Subjects blinded well with appearance of pills factored in, and lack of statistically significant difference in adverse effects between sertraline and placebo groups mitigates disproportionate risk of inference of treatment status	Results analysed in accordance with pre-specified plan, with all baseline or change scores reported	
Lydiard, Stahl [29]	**Some concerns**	**High risk**	**Low risk**	**High risk**	**Low risk**	**High risk**
	Concealment of allocation sequence not discussed, but baseline not significantly difference amongst groups; significance testing not done	Both subjects and investigators blinded, and adherence monitored by pill counts, but no discussion of impact of non-adherence on study participation or statistical analysis, and actual adherence not reported	Whilst around 30% discontinued in each group, proportion similar amongst the groups, and most subjects included in mITT analysis	Whilst subjects were blinded, rapid rate of dose escalation and concomitant greater incidence of side effects meant that inference of allocation to active group could be inferred	Results analysed in accordance with pre-specified plan, with all scores reported	

**Table 3:** Risk of bias table of included non- or quasi-randomised studies, using Risk Of Bias In Non-randomized Studies–of Interventions (ROBINS-I) tool [18].

Non-Randomised Study	Domains of Analysis							
	Confounding	Participant Selection	Classification of Interventions	Unintended deviation from intervention	Missing outcome data	Outcome measurement	Selective reporting	Overall Rating
Itil, Shrivastava [24]	**Serious risk**	**Low risk**	**Low risk**	**Not enough information**	**Serious risk**	**Serious risk**	**Serious risk**	**Serious risk**
	Psychiatric comorbidities not controlled for; also, no baseline demographic or clinical characteristics discussed, with only mention of similarity between the two groups in baseline CGI	Selection of subjects with at least mild-moderate depression (HAMD> = 15) unlikely to introduce significant bias given this is the population most commonly prescribed antidepressants	Intervention well defined and not determined retrospectively	Adherence issues not stated or discussed	Proportion of missing participants greater for fluvoxamine group than other groups and mITT excluded almost 50% of sample	Despite subject blinding, significantly greater proportion of side effects in active groups due to rapid uptitration likely allowed inference of active treatment	No pre-specified plan of analysis stated, and selective reporting with extensive detailing of some measures in table and brief mention of other scores in body	
Van Ameringen, Mancini [25]^a^	**Critical risks**	**Serious risk**	**Low risk**	**Not enough information**	**Low risk**	**Critical risk**	**Low risk**	**Critical risk**
	Lacks control group to control for time and engagement effect, especially when target sample of socially phobic subjects were reviewed regularly; lax exclusion criteria	Inclusion of only generalised social phobia assumes no difference with performance subtype; recruitment from referrals to anxiety disorders clinic likely biases sample towards those with less severe social phobia who are more motivated and/or less impaired to tolerate outside scrutiny of their social phobia that was not factored in analysis	Intervention well defined and not determined retrospectively	Adherence issues not stated or discussed	No missing data	Awareness of intervention makes self-report vulnerable to bias, particularly in sample of socially phobic patients who by definition fears perceived criticism	Analysis occurred according to pre-specified plan, all FQ:Agoraphobia scores reported	
Mancini and Ameringen [26]^a^								**Critical risk**
Finkel, Richter [30]	**Serious risk**	**Serious risk**	**Low risk**	**Not enough information**	**Serious risk**	**Low risk**	**Critical risk**	**Critical risk**
	Inclusion of those with mild cognitive impairment by setting MMSE threshold for inclusion at 24+ and not factoring impact of MMSE scores in analysis	Intentional exclusion of those with treatment-resistant depression introduced bias	Intervention well defined and not determined retrospectively	Even though study assessed adherence by pill count and serum levels, serum levels and pill counts of sertraline not reported or discussed, and criteria for exclusion from study based on pill count was lax	Proportion of participants missing from analysis much higher in nortriptyline group, and only those who completed study was analysed	Subjects blinded, and unlikely to have inferred treatment status as both groups were antidepressants	Pre-specified plan contradictory: stating “for all continuous measures… mean score and mean change score from baseline were computed”, but later stated secondary outcome measures (which include continuous measures) were to only include “changes from baseline”; for POMS, only those who completed study were analysed (vs pre-specified ITT), interaction with time not factored	
Ozdemir, Boysan [31]	**Critical risk**	**Serious risk**	**Low risk**	**Not enough information**	**Low risk**	**Serious risk**	**Low risk**	**Critical risk**
	Psychiatric comorbidities or concurrent medications not controlled for	Inclusion of only inpatient population biases sample towards those with severe MDD and complex comorbidities not factored in analysis	Intervention well defined and not determined retrospectively	Adherence issues not stated or discussed	No missing data	Subjects not blinded, and voluntary status of admission not stated, so highly vulnerable to biases in self-reported measures	Trial analysed in accordance with pre-specified plan, with all POMS scores reported	

^a^Risk of bias assessment results identical.

### Acute Threat/“fear” construct (AT)

4 studies were identified that investigated the effectiveness of 5 antidepressants with FQ:Agoraphobia or FQ:Needle Injury, all of which suffer from significant risk of bias.

The following antidepressants were found to statistically significantly reduce AT:

- Paroxetine at 20-50mg for 8 weeks, with a 46.1% reduction in FQ:Agoraphobia score (from 15.6 +/- 11.9 to 8.4 +/- 10.4), and a 29.1% reduction in FQ:Blood Injury score (from 17.5 +/- 9.5 to 12.4 +/- 10.1, p values not stated), in a sample with DSM panic disorder with or without agoraphobia [19]. Unfortunately, the nature of the values after the mean score was not stated, so it was not possible to derive the standardised mean difference (SMD) between paroxetine and citalopram. Nonetheless, a significant reduction in FQ:Agoraphobia was replicated in a more DSM heterogeneous sample (comprising social phobia, unipolar depressive disorders, panic disorders, specific phobias, GAD, OCD and alcohol dependence) following administration of 10-60mg for 12 weeks (from 11.8+/-10.8 to 5.8 +/- 7.1, p = 0.017) [26]
- Citalopram at 20-50mg for 8 weeks, with a 36.9% reduction in FQ:Agoraphobia score (from 15.7 +/- 12.3 to 9.9 +/- 9.1, p value not stated), and a 46.2% reduction FQ:Blood Injury (from 15.6 +/- 9.9 to 8.4 +/- 10.4, p value not stated), in a sample with DSM panic disorder +/- agoraphobia [19]. A SMD could not be derived for the aforementioned reason.
- Fluvoxamine at 150mg for 12 weeks vs placebo (p<0.05), again in a sample with DSM panic disorder +/- agoraphobia [23]. Unfortunately, there was inadequate data from the study to derive a SMD.

The following antidepressants did not statistically significantly reduce AT:

- Escitalopram at 10-20mg for 12 weeks in a sample with DSM specific phobia [21], from 3.0 (SD = 2.4) to 1.2 (SD = 1.1) (p value not stated).
- Nefazodone at 200-600mg for 12 weeks in a sample with heterogeneous DSM diagnoses comprising generalised social phobia, unipolar depressive disorders, panic disorder, specific phobia, GAD, OCD and alcohol dependence, from 9.0 (SD = 7.8) to 6.4 (SD = 6.5) (p = 0.09) [25]

### Potential Threat “anxiety” (PT)

3 studies were identified that investigated the effectiveness of 3 antidepressants with POMS:Tension, all of which suffered from significant risk of biases.

The following antidepressants statistically significantly reduced PT:

- Desvenlafaxine at 100mg or 150mg for 12 weeks in a sample with vasomotor symptoms and no DSM diagnoses vs placebo, with a 4.1 reduction in POMS:Tension score from a baseline of 9.3 (SD = 6.7) for 100mg dose, and a 3.9 reduction from a baseline of 9.0 (SD = 7.2) for 150mg dose (p<0.05) [20]. From the values provided for these dosages and for placebo, a SMD vs placebo of 0.26 for 100mg and 0.22 for 150mg were derived.
- Fluoxetine at 20mg for 6 months in a sample with PMS and no DSM-IIIR diagnoses vs placebo during the luteal phase only (p<0.005), when PT was found to be higher than during the follicular phase (T scores of 56.6 +/- 3.3 vs 40.5 +/- 3.6) [22]. From these values and those for placebo, a SMD of 1.1 vs placebo could be derived.
- Venlafaxine at 75-150mg for 8 weeks appeared to significantly reduce PT as well in a sample of DSM MDD where comorbid anxiety, phobic or personality disorders not excluded, but unfortunately no significance testing was performed to guide interpretation of the reduction in POMS:Tension score from 23.68 +/- 4.88 to 9.6 +/- 3.93 [31].
- Sertraline at 50mg or 150mg for 12 weeks appeared to significant reduce PT in a sample with DSM MDD that is potentially heterogeneous in nature due to exclusion criteria being acute or chronic organic mental disorder and “clinically significant psychiatric illness (including) active suicidality” by 5.9 (confidence interval not given) [30]. However, significance testing was limited to comparison with nortriptyline (p = 0.01).

### Loss

7 studies were identified that investigated the effectiveness of 5 antidepressants wi8th POMS:Depression.

The following antidepressants were found to statistically significantly reduce Loss:

- Sertraline at 50/100/200mg for 6 weeks vs placebo (p<0.01) reduced POMS:Depression score by 0.1 (SD = 0.7) from a baseline of 2.7 (SD = 1.1) in a potentially heterogeneous sample of DSM MDD where comorbid diagnoses including anxiety, phobic, obsessive and personality disorders weren’t excluded [28]. From these values and those for the placebo group, a standardised mean difference vs placebo of 0.39 was derived. A reduction was replicated at 50-200mg for 8 weeks in a similar sample of those with DSM MDD where comorbid mild-moderate GAD or personality disorders weren’t excluded, but the reduction was much greater, by 1.0 (SE = 0.08) from a baseline of 1.9 (SE = 0.08) [29]. This was equivalent to a SMD of 0.56 vs placebo.

However, when the sample comprise those with MDD where comorbid mild neurocognitive disorder with MMSE score > = 23 or personality disorders were not excluded, the reduction from 1.5 (SD = 0.67) to 1.2 (SD = 1.13) did not reach statistical significance (p value not stated) [27].

- Desvenlafaxine at 100mg or 150mg for 12 weeks vs placebo for those with VMS but no DSM comorbidities, by 5.5 from a baseline of 8.3 (SD = 8.6) for 100mg and by 4.5 from a baseline of 8.2 (SD = 11.1) for 150mg [20]. From these values and those for the placebo group, a SMD of 0.22 for 100mg and 0.35 for 150mg vs placebo could be derived.
- Fluvoxamine at 50-200mg for 4 weeks in a heterogeneous Research Diagnostic Criteria MDD sample where the only exclusion was substance dependence, but statistical significance only reached at week 3 (p = 0.028, vs p = 0.6 at week 4) [24]. Unfortunately, there was inadequate data available from the study to derive a SMD.
- Fluoxetine at 20mg for 6 months vs placebo (p<0.005) in a sample with PMS but no DSM-IIIR comorbidities during the luteal phase only, when POMS:Depression was greater than during the follicular phase (T scores of 59.6+/-4.6 vs 44.8 +/- 5.2) [22]. From these values and those for placebo, a SMD of 0.93 vs placebo could be derived.
- Venlafaxine at 75-150mg for 8 weeks also reduced POMS:Depression in a sample with DSM MDD where comorbid diagnoses including anxiety, phobic and personality disorders were not excluded, seemingly significantly by 70.2% from 37.16 +/- 11.36 to 11.12 +/-9.11; however, significance testing was not performed [31].

## Discussion

### Key conclusions

This is the first systematic review to investigate the effectiveness of current generation antidepressants using the RDoC paradigm. The review revealed that a remarkably limited proportion of studies included self-report psychometric measures proposed by Watson, Stanton and Clark [10] to correlate with the NVS constructs of AT, PT and Loss. The 13 studies identified used a total of three out of the seven instruments, despite the fact that most of them were developed a number of decades ago, likely reflecting the inherent primacy of DSM-validated measures in psychiatric research. The articles investigated 8 current generation antidepressants, and found that the majority were effective in reducing the severity in the construct(s) concerned (i.e. paroxetine, citalopram and fluvoxamine for AT, fluoxetine, desvenlafaxine and sertraline for PT, and sertraline, fluvoxamine, fluoxetine & desvenlafaxine for Loss), although the strength of this conclusion was limited by the high risk of bias within included studies.

### Antidepressants lacking evidence of effectiveness along particular RDoC constructs

This review found a lack of evidence of effectiveness for 2 antidepressants: escitalopram and nefazodone for AT. The finding for nefazodone is particularly interesting because the study concluded that nefazodone was effective for generalised social phobia, based on a range of other psychometric measures such as the State-Trait Anxiety Inventory (STAI) that reported statistically significant improvement 25). However, discriminant validity is a pervasive issue with psychometric instruments for anxiety and depression [10] with psychometric instruments–such as the STAI–often measuring general distress/negative affectivity as opposed to more specific constructs of anxiety, fear or depression. Van Ameringen, Mancini [32] studied nefazodone again using a more rigorous randomised placebo-controlled design with a much larger sample size and a completely different set of outcome measures, and concluded that nefazodone was in fact ineffective for generalised social phobia. In light of trazodone’s comparatively poor receptor affinity for serotonin transporter relative to those antidepressants found to be effective for AT [33], and the established role of the serotonin system in threat regulation [34, 35], this finding is not surprising.

Unlike trazodone, established evidence contrary to this review’s finding of a lack of effectiveness for escitalopram is based on psychometrics that measure agoraphobia like FQ:Agoraphobia (e.g. Panic and Agoraphobia Scale) [36–38]. Thus, the findings of Alamy, Wei [21] likely reflects risk-of-bias issues within the study, rather than the principle of assessing psychopathology in terms of behavioural-psychological constructs such as AT versus more heterogeneous DSM constructs such as “panic disorder”.

The lack of studies investigating other antidepressants on other constructs unfortunately preclude further consideration of the constructs’ biological underpinnings.

### Extent of effectiveness

A few studies provided adequate data to allow SMDs vs placebo to be derived. These demonstrated that for PT, fluoxetine was very effective whilst desvenlafaxine was weakly effective (SMD = 1.1 vs 0.22–0.26 in POMS:Tension, respectively). For Loss, fluoxetine was similarly very effective whilst sertraline was moderately effective and desvenlafaxine again weakly effective (SMD = 0.93 vs 0.39–0.55 vs 0.22–0.26 in POMS:Depression, respectively).

Whilst the finding of effectiveness of antidepressants for both depression and anxiety is hardly surprising, the lack of overlap in confidence intervals amongst the antidepressants concerned is. Whilst caution is needed to interpret this given the low reliability of this finding (discussed below), it is worth noting that recent network meta-analyses of GAD [39] and MDD [40] found overlapping confidence intervals amongst all antidepressants such that the present review’s findings are not inconsistent with those reported.

It is tempting to consider desvenlafaxine’s comparatively poorer effectiveness to fluoxetine and sertraline in these constructs as a reflection of the effectiveness of targeting noradrenaline receptors in the treatment of PT and Loss. However, pharmacological studies serve as a reminder that the difference between SSRIs and SNRIs lie in their affinity for the noradrenaline transporter relative to the serotonin transporter, not in their absolute affinities for either receptors [33, 41–43]; for instance, sertraline is a more potent inhibitor of noradrenaline transporter than both venlafaxine and desvenlafaxine, and only has slightly lower affinity than duloxetine, not to mention its higher absolute affinity for the dopamine transporter.

Further, it is important to note that with regards to GAD, the DSM diagnosis most similar to the RDoC construct of PT, no studies to date have investigated the effectiveness of desvenlafaxine for GAD. Thus, the fact that a testable hypothesis for the effectiveness of an antidepressant for a particular disorder was able to be generated from this review, based on the results of a study investigating a sample with purely vasomotor symptoms and no DSM diagnoses, suggest the potential utility from both research and clinical perspectives of transdiagnostic constructs.

### Risk of bias in included studies

Unfortunately, the quality of these studies were uniformly poor, and all were prone to significant risk of biases affecting the reliability of their findings and conclusions. However, this must be considered in the context of the wider clinical trial literature. In the aforementioned network meta-analyses, one identified only 18% of the 522 included trials as having a low risk of bias with the vast majority rated as moderate (73%) [40], whilst the other rated 84% of the 89 trials they included as a high risk of bias in at least one of the domains [39], meeting the criteria established by the authors of the tool for an overall high risk of bias. Similarly, in a systematic review of the efficacy and safety of adjunctive antidepressants in schizophrenia [44], of the 82 trials included, 60% met criteria for an overall high risk of bias. Again, in a systematic review of prophylactic antidepressant treatment following acute coronary syndrome [45], of the 6 studies included, all were rated as having a high risk of bias.

Complicating matters is the fact that assessments of risk of bias is subjective, and despite the development of tools and associated guidance documents to facilitate in the analysis, it has been found that 45% of subfertility trials included in more than 1 Cochrane review received differing risk of bias judgements from different groups of authors, with greater agreement in random sequence generation (71%) and incomplete outcome data (79%) and less agreement in blinding (35%) [46]. A strength of this study is the use of the RoB2 and ROBINS-I risk of bias assessment tools for analysing randomised and non-randomised trials, respectively, due to their comprehensive scope and detailed guidance for grading levels of risk.

It can thus be said that risk of bias issues are not limited to the articles identified within this review, but pervade the clinical literature at large. Hence, a useful rule of thumb for interpreting findings of any study or review is to be ever-vigilant and thoughtfully consider them in the context of its methodology and one’s clinical question of interest. Authors of systematic reviews can facilitate readers by being more overt about their risk of bias judgements, for example by moving the risk of bias table from the appendix or supplementary material to the body of the paper, and by providing the underlying rationale in addition to the summary judgement within each domain.

Nevertheless, it was noteworthy that the few times the same antidepressant was investigated within the same construct, the findings were replicated by different investigators (i.e. paroxetine and AT, sertraline and Loss). This is even more remarkable considering the heterogeneous nature of the samples’ DSM diagnoses. Whilst a caveat must be given since the association between sertraline and loss in an older population with likely mild cognitive impairment did not reach statistical significance, the results nonetheless suggest further research is warranted to corroborate the findings of this review with the view to furthering the evidence base with respect to transdiagnostic constructs.

### Construct validity of “potential threat” and “loss” in the negative valence domain

This review identified 3 studies that investigated outcomes for specific antidepressants along multiple constructs; all were along both PT and Loss constructs. 2 were in samples with PMS/VMS, and both reported similar scores with significant overlap in 95% confidence intervals in both measures at baseline and endpoint [22], during follicular and luteal phases [20], and they reported similar levels of improvements in both measures with antidepressant treatment. The third study [31] reported baseline POMS:Depression that appeared to be higher than POM Tension, but a small overlap in confidence interval still existed; endpoint POMS measures after antidepressant were similar with considerable overlap in confidence intervals. There is thus a need to further study the ability of POMS-Depression and POMS-Tension to differentiate amongst different phenomenological manifestations of the pathophysiological processes underlying PMS/VMS and MDD, or of antidepressant treatment.

Whilst the 65 adjectives that constitute POMS were found to cluster into 6 distinct factors/subscales, concerns had been raised that the original list of adjectives from which the factors were derived were created with these 6 factors in mind [47]. Moreover, factor analyses of categorical psychiatric disorders have failed to demonstrate that MDD and GAD (the DSM disorder that best represent the Response to PT construct) belong to different factors, either within psychiatric samples [48–50], community samples [51–53], child and adolescent samples [54–56] or cross-cultural samples [57]. This phenomenon persisted when factor analysis occurred on a symptom level, with “internalising disorders”–a factor which influenced RDoC’s NVS [58]–consistent with other factor analyses, found to be better described by a “fear” factor and a “general distress” factor [59].

Further, the neurobiological substrates subserving responses to PT and loss appear to share considerable overlap. Indeed, it is still not possible to differentiate between GAD from MDD based on a review of a multimodal neuroimaging evidence to date although the same study found a correlation between limbic/paralimbic activity and the combined sample including healthy controls based on levels of general distress [60]. Of course, similarity between the DSM diagnoses of MDD and GAD and the RDoC constructs of response to Loss and PT do not constitute equivalence, but evidence using POMS:Tension and Depression also suggest a convergence in correlation between PT and Loss on the one hand, and impaired activity of the anterior cingulate cortex [61, 62] and amygdala [63], as well as reduced GABA-A receptor binding in the posterior cingulate cortex and left superior frontal regions [64] on the other. Increasing evidence that physiological paradigms such as startle response are a reliable differentiator of fear or distress [65] suggests that a more biologically-grounded conceptualisation of NVS constructs is simply AT vs non-AT. This is further supported by evidence implicating only serotonin (and not dopamine or noradrenaline) in the startle response [66], as well as meta-analytic studies reporting reboxetine to be effective in MDD [40], but not panic disorder [67].

The idea of a NVS “distress” construct is actually not new. The initial NIMH workgroup responsible for creating RDoC initially proposed that the NVS comprise of “distress”, “fear”, and “anger” constructs [1]. However, an expert workshop convened to discuss these proposals instead decided to split the “distress” construct into response to “PT”, “ST” and “loss” because the former was found to be too “vague and diffuse”, and deemed the revisions better to “accommodate a wide range of experiences and situations that logically fall under the Construct” [1]. Interestingly, the workshop conceded “uncertainty about whether ST should be considered as a separate Construct, or as variations… that impact the circuits involved in AT and potential harm”, but “decided to include [it]” with the caveat that “further clarification is needed” [1]. There did not seem to be much debate about the “loss” construct, as it was “the most frequently nominated Construct of the pre-workshop survey”; depression was considered the “sustained” response to loss [1].

However, it must be said that the workgroup acknowledged the list of Constructs created was “not intended to be definitive or all-inclusive” [1]. Similarly, when pondering the future in light of the body of research that led to the development and publication of the DSM-III, Spitzer wrote that it was “only one still frame in the ongoing process of attempting to better understand mental disorders” [68]. A similar sentiment was cited by Kozak and Cuthbert [58] to warn against the reification of RDoC constructs that had befallen DSM-III diagnostic categories.

### Psychometric score interpretation

When validated against a representative community sample, normal POMS:Depression score was 7.5 (SD = 9.2) for men and 8.5 (SD = 9.4) for women, and normal POMS:Tension score was 7.1 (SD = 5.8) for men and 8.2 (SD = 6.0) for women [69]. For FQ, the normal score in a representative community sample was 7.9 (SD = 7.1) for men, 14.8 (SD = 8.5) for women, and 11.8 (SD = 8.6) overall [70]. Therefore, with the exception of the only study performed in an inpatient population [31], all the other studies identified by this systematic review reported mean baseline values of their subjects that were within the “normal” range.

Given that the inclusion criteria for these study were based on clinical judgements according to the DSM or related psychometric instruments, this finding raises questions about the relationship between observer and self reports as well as the relationship between DSM diagnoses and RDoC.

With regards to the former, whilst a number of questions about the validity of HAMD as a measure of depression severity have been raised [71], it should be pointed out that POMS asks subjects to rate how strongly a word/phrase describes them over a period of time [12]. However, none of the studies identified reported the timeframe, making it more difficult to interpret the meaning of these results although the low values suggest a timeframe much shorter than that used by the instruments used by clinicians. Nonetheless, underreporting of mood had been described in the literature due to response bias motivated by perceived social desirability [72, 73]. Thus, for RDoC research to be clinically useful, considering expanding the “self-report” unit of analysis to include observer-rated elements of such self-report is warranted.

With regards to the latter, it could be observed from the 3 studies included in this systematic review that reported baseline FQ:Agoraphobia scores that their samples consisted primarily of generalised social phobia [25, 26] and panic disorder [19]. Given the FQ:Agoraphobia subscale assesses the severity of the respondent’s behavioural avoidance and not affective distress, these samples’ normal scores can be seen as a reflection of DSM’s heterogeneous criteria for severity, namely “a) persistent concern… or b) worry about the implications (of the [panic] attack)” versus “significant change in behaviour” for panic disorder, and “the feared social or performance situations are avoided or else are endured…” for social phobia. In light of evidence that generalised social phobia correlate with “distress” disorders whereas specific social phobia correlate with “fear” disorders, it is thus more understandable why samples of the former [25, 26] scored within the normal range but much lower that of the latter [19], whose mean baseline scores were higher than the normative means. Further, it suggests the need to further study the potential for RDoC’s dimensionally-based constructs to differentiate amongst not just normal and pathological, but amongst different types of pathology as well.

Another observation that can be made from the 3 studies that measured FQ:Agoraphobia is the effectiveness of antidepressants along this dimension in spite of the “normality” of the scores. This finding is suggested by results of a recent meta-analysis concluding that antidepressants are effective across all severity levels of anxiety disorders [74], and reinforces earlier findings that antidepressants can reduce attentional vigilance to threat in healthy volunteers [75]. These findings are consistent with the dimensional conceptualisation of psychobehavioural constructs spanning normality to pathology. Further study could shed light on whether antidepressants have the potential to be used as emotional nootropic agents.

#### Other recommendations for further study

Novel psychometric measures need to be designed if meaningful intervention trials in psychiatry are to be conducted using the RDoC framework, especially as it relates to antidepressants and NVS. However, regardless of a one’s intentions with regards to RDoC, researchers in psychiatry should be more cognisant of what psychometric instruments (particularly those that are frequently used) actually measure and the extent to which valid interpretations could be made from them, especially as it relates to the instruments’ underlying latent construct(s).

## Conclusion

In conclusion, this systematic review found only 13 studies of SSRI or later generation antidepressants that used outcome measures found by Watson et al to correlate closely with the NVS constructs of AT, PT and Loss. Paroxetine, citalopram and fluvoxamine were found to be effective for AT; fluoxetine, desvenlafaxine and sertraline for PT; and sertraline, fluvoxamine, fluoxetine and desvenlafaxine effective for Loss. SMDs derived from studies that reported the necessary data revealed that for PT, fluoxetine was superior to desvenlafaxine (SMD = 1.1 vs 0.22–0.26, respectively), and for Loss, fluoxetine was superior to sertraline, and both were superior to desvenlafaxine (SMD = 0.93 vs 0.39–0.55 vs 0.22–0.26, respectively).

The clinical utility for transdiagnostic constructs were suggested by the lack of evidence found for nefazodone in AT, in addition to replication of effectiveness for paroxetine in AT and sertraline in Loss by different author groups within heterogeneous DSM samples. The clinical utility of dimensional constructs were similarly suggested by differential baseline FQ:Agoraphobia scores between samples of DSM generalised social phobia and DSM specific social phobia. The validity of PT and Loss as separate constructs is questioned, consistent with a developing body of evidence suggesting significant overlap between the two. However, the strength of these findings were limited by the high risk of biases from included studies.

Finally, this review postulated two hypotheses that, if true, would support the clinical utility of both transdiagnostic and dimensional characteristics of RDoC constructs: specifically, that desvenlafaxine was effective for DSM GAD, and antidepressants are effective for regulating affect, cognition or behaviour in those with no DSM disorders.

## Supporting information

S1 Checklist (DOC)
